# Novel limbal dermoid surgery for visual acuity and cosmesis improvement: A 7-year retrospective review

**DOI:** 10.1371/journal.pone.0286250

**Published:** 2023-06-02

**Authors:** Jinho Jeong, Gabriel M. Rand, Ju-Yeun Lee, Ji-Won Kwon

**Affiliations:** 1 Department of Ophthalmology, Jeju National University College of Medicine, Jeju, Korea; 2 Department of Ophthalmology, Columbia University Medical Center, New York, NY, United States of America; 3 Department of Ophthalmology, Myongji Hospital, Hanyang University College of Medicine, Goyang, Korea; Aravind Eye Hospital and Post Graduate Institute of Ophthalmology, INDIA

## Abstract

**Background:**

To report a long-term outcome of the novel combined surgical method of complete excision, corneal tattooing, and a sutureless limbal conjunctival autograft for limbal dermoid.

**Methods:**

All patients who were referred to our clinic for limbal dermoid, and underwent a combined surgery of complete excision, corneal tattooing, and a sutureless limbal conjunctival autograft were retrospectively reviewed. The surgery was performed by one surgeon, and all clinical information was obtained during a seven-year follow up period. In all patients, surgical outcomes of cosmesis, best corrected visual acuity (BCVA), spherical equivalent (SE), and corneal/ocular astigmatism were obtained and compared preoperatively and postoperatively.

**Results:**

During seven years, 24 patients (24 eyes) with limbal dermoid were finally enrolled. The mean age was 10.1±8.9 years old. The surgery resulted in an improved appearing ocular surface in all cases without any complications. There was no statistical difference in BCVA, corneal and ocular astigmatism between preoperatively and postoperatively (p = 0.231, 0.156 and 0.475, respectively). The mean SE was 0.12±3.19D preoperatively, and -0.21±3.02 D postoperatively with statistical significance (p = 0.037). Mean follow up period was 54.50 ± 15.62 months.

**Conclusions:**

Based on the results of this study, our innovative surgical method which includes complete excision with corneal tattooing and limbal conjunctival autograft can be a simple and safe procedure that achieves long standing cosmesis with limbal dermoids.

## Introduction

A limbal dermoid is a solid choristoma of ectodermal and mesodermal origin that resembles epidermal/dermal tissue [1]. It often occurs in the inferotemporal quadrant of the cornea as a solitary lesion, and its growth is usually limited. The Mann grading system classifies limbal dermoids by their size and depth of involvement. Grade I is smaller than 5mm in diameter and is superficial, Grade II is larger and/or involves the deeper cornea anterior to Descemet’s membrane, and Grade III involves the full corneal thickness with extension into the anterior chamber and is very rare [2]. Historically, surgery had been reserved for the treatment of dermoid-induced amblyopia; however, as the psychosocial impact of these lesions has gained recognition and as treatment modalities improved, cosmesis has become an accepted surgical indication [3–5].

Complete excision alone is sufficient for small Grade I lesion. For larger grade lesions, complications of complete excision may include persistent epithelial defects, dellen, peripheral corneal vascularization and scarring [6]. Focal limbal stem cell deficiency may contribute to some of these adverse effects [7]. Lamellar keratoplasty can be used for higher grade lesions, however it requires donor tissue, is technically more complex, and carries the risk of graft rejection [8–10]. We were interested in the pursuit of a simple technique that provided aesthetic results with minimal complications. We previously reported two years of results on our use of a novel technique that includes dermoid excision, corneal tattooing, and sutureless limbal conjunctival autografting for Grade I limbal dermoids [4]. The objective of the procedure is to remove dermoid completely while preserving a normal corneoscleral plane and to mask the residual corneal opacity with tattooing. In the present study, we report on 24 new cases from a 7-year period that include limbal dermoids which were less than grade II.

## Material and methods

### Ascertainment of the participants

A retrospective chart review was conducted on 24 eyes of 24 patients who underwent complete dermoid excision, corneal tattooing, and sutureless limbal conjunctival autografting in Myongji Hospital Hanyang University College of Medicine between May 2011 and June 2018. All patients with a diagnosis of limbal dermoid were referred to our clinic. Inclusion criteria were apparent corneal disfigurement due to limbal dermoid, and the dermoid size larger than 3 mm, dermoid grade less than Grade II confirmed by anterior segment optical coherence tomography (anterior segment OCT, CIRRUS™ HD-OCT 6000, Zeiss, Dublin, CA). Exclusion criteria were patients with previous ocular operation history, other ocular comorbidities like retinal disease, and the cases with post-operative follow-up less than 3 years.

**Fig 1:**
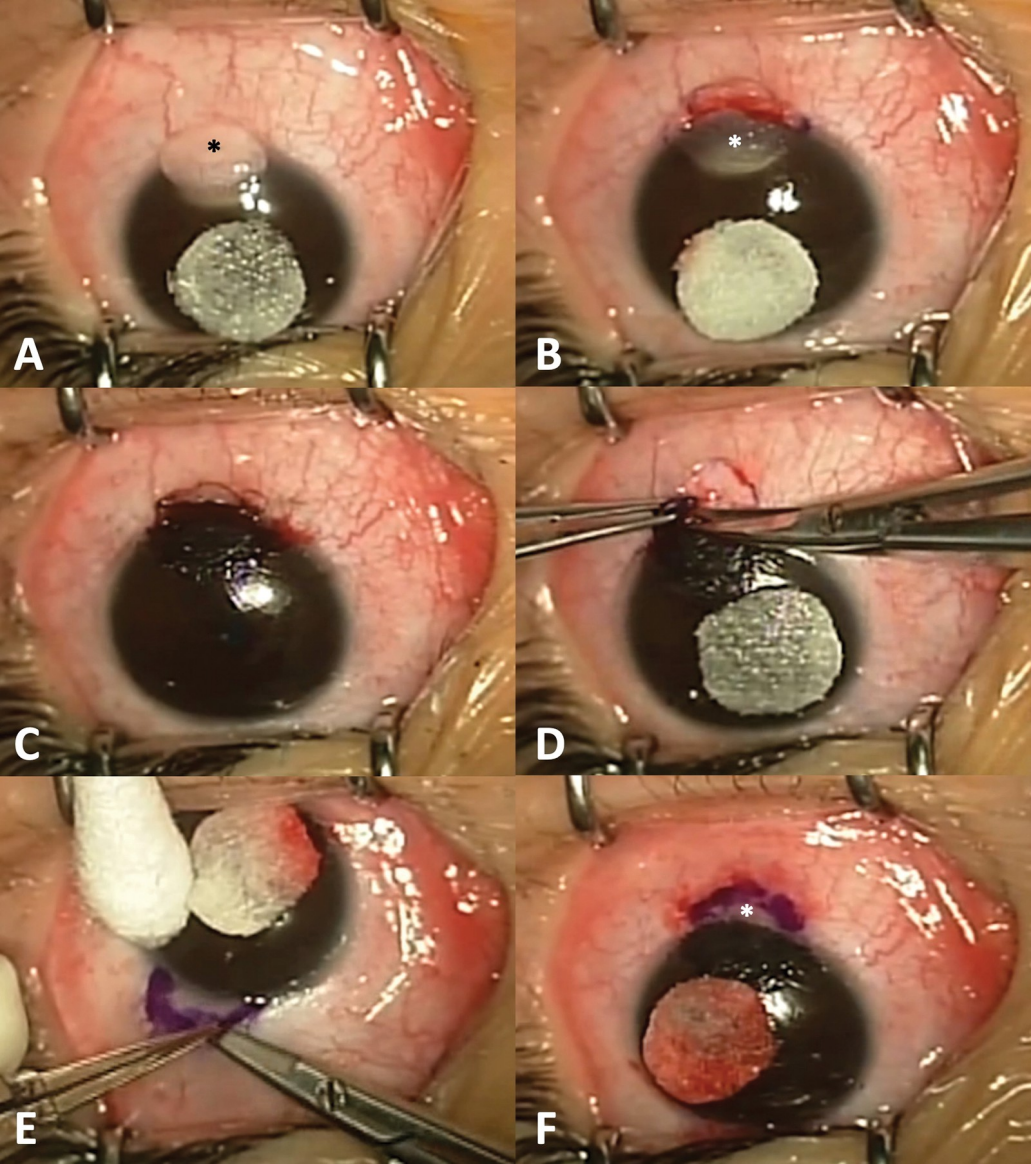
The surgical procedure for complete excision, corneal tattooing, and sutureless conjunctival limbal autograft (Case #3). The limbal dermoid is visualized inferiorly (*) (A); The corneal portion of the dermoid is excised with a residual stromal opacity(*) (B); The opacity is tattooed through anterior stromal puncture (C); The conjunctival portion of the dermoid is excised (D); A superior conjunctival limbal graft was harvested(*) (E); The graft is adhered over the conjunctival defect with fibrin glue(*) (F).

**Fig 2:**
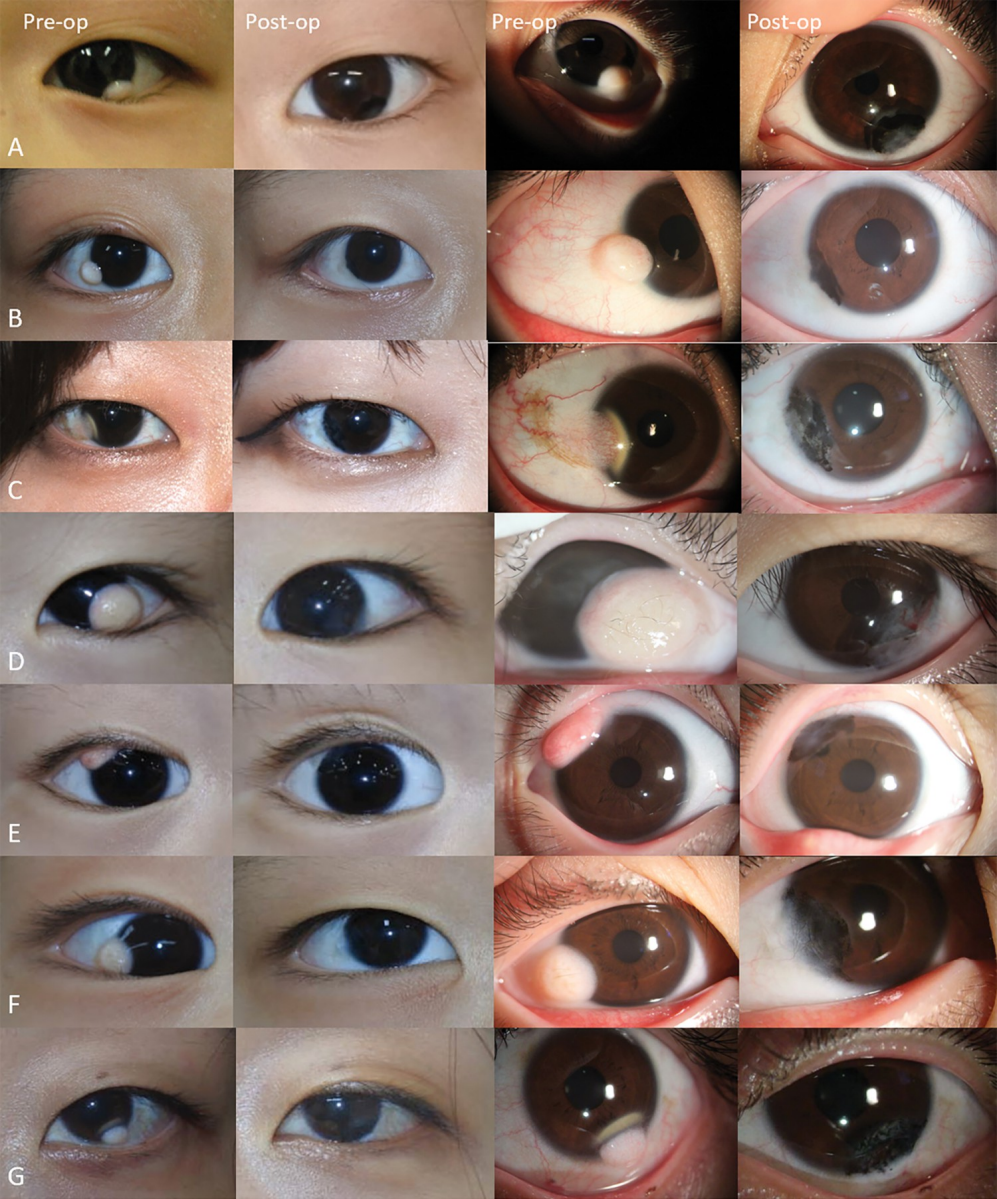
Pre- and post-operative external photographs and high magnification slit lamp photographs of limbal dermoid patients. A: Case #5. A 5-year-old female with a inferotemporal limbal dermoid. B: Case #8. An 11-year-old female with a inferotemporal limbal dermoid. C: Case #9. A 22-year-old female with a temporal limbal dermoid with vascularization. D: Case #13. A 3-year-old female with a inferotemporal limbal dermoid. E: Case #18. A 3-year-old male with a superotemporal limbal dermoid. F: Case #19. A 3-year-old male with a inferotemporal limbal dermoid. G: Case #20. A 31-year-old female with a inferotemporal limbal dermoid. All the postoperative photos were taken 3 years after surgery.

**Fig 3:**
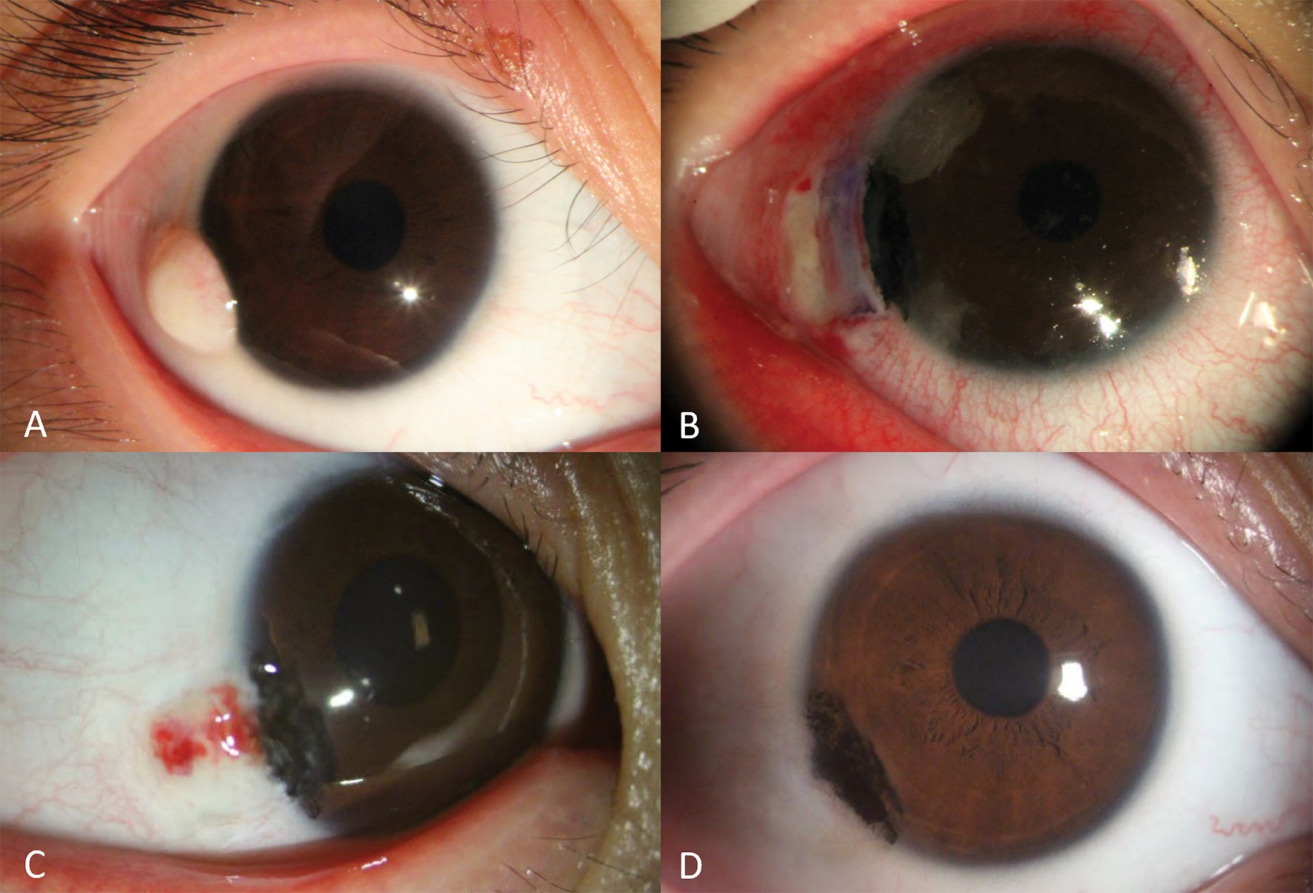
Serial changes of anterior segment photography of case #8. The inferotemporal limbal dermoid is seen(A), and on postoperative day 1, grafted conjunctiva is clearly seen(B). After 3 weeks, no injection or resolution of subconjunctival hemorrhage was observed. The grafted conjunctival margin was faintly visible (C). No conjunctival neovascularization or pseudo-pterygium was found, indicating good cosmetic results. postoperative 3 years(D).

**Fig 4:**
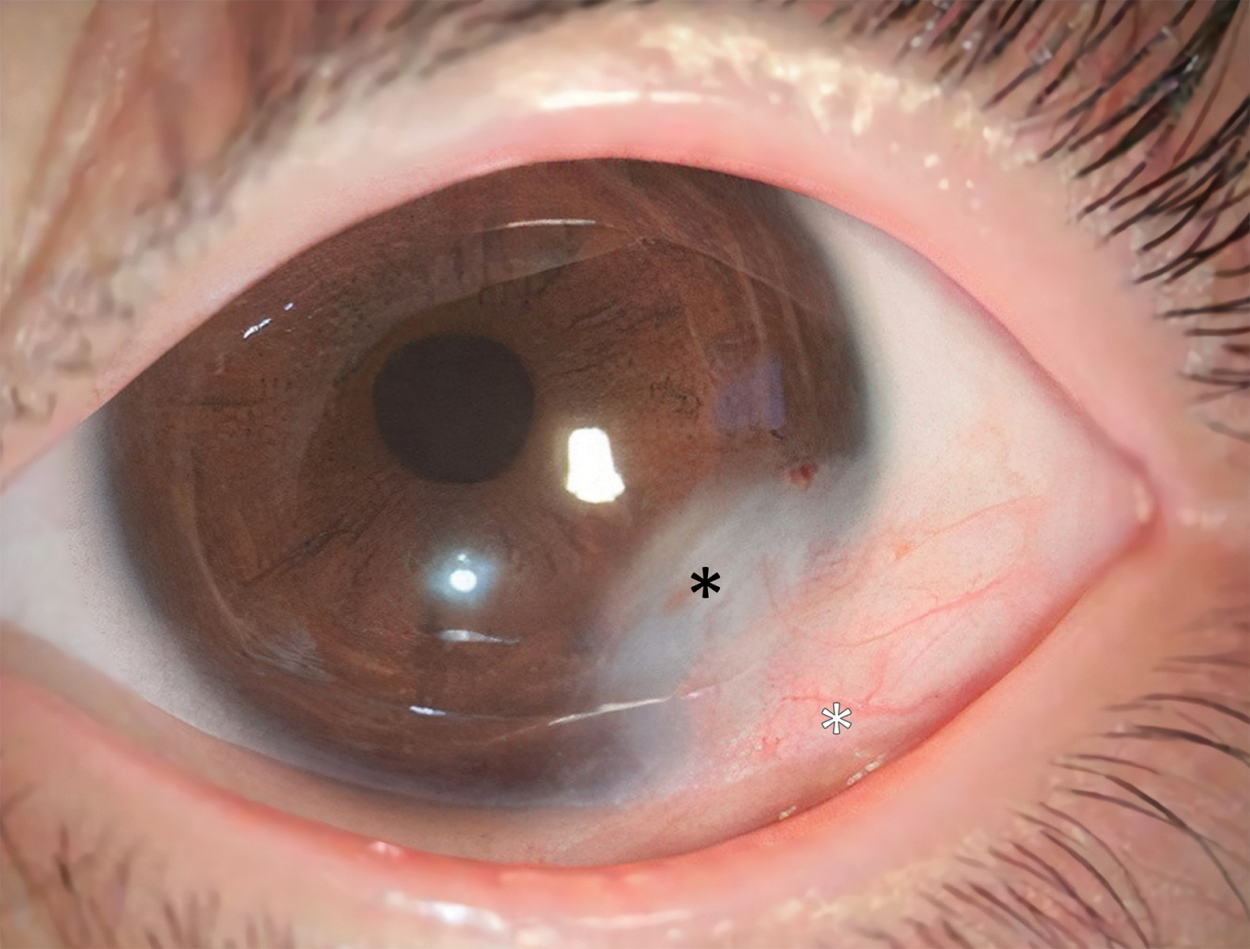
Postoperative photos of a simple inferotemporal dermoid excision. In this photo, we can see prominent corneal opacity(*) and conjunctivalization(white *) with many vessels of adjacent conjunctiva which makes the eye look injected. The surgery was done at the other hospital.

The goal of the surgery was to improve cosmesis and vision. All information was routinely collected as a part of standard care at the clinic at Myongji Hospital Hanyang University College of Medicine. The study protocol was in accordance with the Declaration of Helsinki and was approved by the institutional review board of Myongji Hospital, Hanyang University College of Medicine (2019-01-015-003). Written informed consent was obtained from all participants’ parents or legal guardians, and all data were anonymized. The individual pictured in Figs 1–4 has provided written informed consent as outlined in PLOS consent form to publish their image alongside the manuscript.

### Outcome measurements

In all patients enrolled in this study, family and past medical histories, review of systems, logMAR best corrected visual acuity (BCVA), spherical equivalent refractive error (manifest refraction or retinoscopy), automated keratometry (KR-800, Topcon, Tokyo, Japan), topography(Pentacam, Oculus, Wetzlar, Germany), anterior segment OCT and slit lamp biomicroscopy examination were performed at the initial visit. The patients routinely visited the clinics on postoperative week 1, 2 and months 1 and 3. Afterwards they were evaluated at 6 months intervals. The primary outcome was cosmetic appearance which can be assessed by the reader by external photographs. Secondary outcomes included changes in logMAR BCVA, spherical equivalent (SE), corneal astigmatism measured by each automated keratometry, and ocular astigmatism measured by manifest refraction.

### Surgical procedure

One surgeon (JWK) performed all of the procedures. All procedures were conducted under general anesthesia. As the tattooing ink precipitates in tissues that are de-epithelized and are difficult to remove, the corneal part of the dermoid is excised first to prevent this complication. The border of the limbal dermoid was marked at a shallow depth using a #69 Beaver blade (Fig 1A). The corneal section of the dermoid was held by 0.12 forceps and carefully excised using the Vannas scissors (Fig 1B). After confirming the residual corneal opacity with the room light (Fig 1B), tissue-marking dye (Bradley Products Inc., Minneapolis, MN, USA), which had been sterilized in an autoclave at 134°C for 6 minutes before surgery, was used for masking the residual white opacity [4, 5]. With the bevel up, the punctures were carefully made so as not to perforate the cornea, into the opacified anterior stroma horizontally to the corneal plane using a 1-mL disposable syringe with a 30-gauge needle, creating a relatively long puncture canal. Because the corneal surface was obscured by the dye after several punctures, the spread of the dye was confirmed by vigorous irrigation with a balanced salt solution (BSS) (Fig 1C). After confirming that the corneal tattooing was sufficiently complete and the color of the opacified cornea changed to black, excision was performed on the conjunctival part of the dermoid using Vannas scissors (Fig 1D). To measure the size of the conjunctival defect with a caliper, the superior conjunctiva, including the limbal margin for the autograft, was marked with gentian violet of the same size. Lidocaine solution was injected into the marked subconjunctival space to separate conjunctival epithelium and underlying Tenon tissue to make dissection easier. The limbal conjunctiva was carefully harvested, similar to the pterygium operation (Fig 1E) and the graft was immediately transferred onto the bare sclera. Fibrin glue (Tisseel; Baxter Inc., Deerfield, IL) was then applied under conjunctival graft giving firm adhesion between the sclera and the autografted conjunctiva (Fig 1F). The ocular surface was covered with a bandage contact lens (Johnson and Johnson Acuvue 1 day, -0.50 Diopter, 14.2 mm in diameter, 8.5 mm in base curve), and patients received 1.5% levofloxacin (Cravit; Santen Pharmaceutical Company, Osaka, Japan) and 1% prednisolone acetate (Pred Forte; Allergan, Irvine, CA, USA) four times per day for 3 weeks. The bandage contact lens was removed at postoperative week one or two.

### Statistical analysis

Continuous variables were presented as mean ± standard deviation (SD). The Paired Wilcoxon signed-rank tests were used to test preoperative and postoperative differences in quantitative outcome variables at the postoperative 3-year visit. Statistical analyses were performed using SPSS 25.0 for Windows (SPSS, Chicago, IL). A p-value of less than 0.05 was considered statistically significant.

### Data availability

The datasets analyzed during the current study are available from the corresponding author on reasonable request.

## Results

### Demographics

Among the 27 patients (27 eyes) enrolled during the seven-year period, two patients were excluded for follow up loss and one patient was excluded for having other ophthalmologic disease. At last, 24 patients (24 eyes) were finally enrolled in this study (Table 1). The mean age at presentation was 10.1±8.9 years old (range 1 to 31 years). The male to female distribution was 8 and 16, respectively. All cases were classified less than Grade II. The average total diameter of the limbal dermoid was 6.3±4.9 mm. All cases were unilateral with equal involvement of right and left eyes. Dermoid location was inferotemporal in 16 eyes, temporal in 6 eyes, superotemporal in one eye, and inferior in one eye. Four cases had an associated head or neck dermatologic abnormality. The preoperative appearance of a number of the patients can be seen in Fig 2. Mean follow-up period was 54.50 ± 15.62 months ranging from 41 months to 94 months.

**Table 1:** Demographic and preoperative and postoperative vision data of 24 limbal dermoid cases.

Case No.	Gender	Age (years)	Eye	Site	Lesion Size (horizontal, mm)	Follow-up (months)	Dermatologic Abnormality	Preoperative BCVA (logMAR)	Postoperative BCVA (logMAR)
1	F	2	L	IT	4	89		0	0
2	F	6	R	IT	5	89	Facial nevus sebaceous	0	0
3	M	9	L	IT	7	39	Orange papule on neck	0	0
4	F	4	L	T	4	38		0.3	0.2
5	F	5	L	IT	5	38		0.3	0.3
6	M	5	R	IT	5	37		0.2	0
7	M	6	L	IT	5	36		0	0
8	F	11	R	IT	4	36	Right ear skin tag	0	0
9	F	22	R	T	7	36		0	0
10	F	18	R	T	6	61		0.2	0.2
11	F	13	R	IT	4	36		0	0
12	F	20	R	T	7	36		1.0	1.0
13	F	3	L	IT	8	55		0.7	0.5
14	M	6	L	T	6	49	Goldenhar syndrome	0.7	0.7
15	F	2	R	IT	10	56		—	—
16	M	27	L	IT	4	52		0.2	0.2
17	F	8	R	IT	5	64		0.7	0.4
18	M	3	R	ST	5	60		0.5	0.5
19	M	3	R	IT	6	57		0.7	0.7
20	F	31	L	IT	3	55		0	0
21	M	1	L	IT	5	36		—	—
22	F	9	L	I	5	51		0	0
23	F	5	L	T	28	37		0	0
24	F	27	L	IT	3	42		0	0

No.: number, F: Female, M: Male, L: Left, R: Right, IT: Inferotemporal, T: Temporal, ST: Superotemporal, I: Inferior, BCVA: Best Corrected Visual Acuity

### Primary outcomes: Cosmesis and complications

There were no intraoperative complications such as extensive hemorrhage or corneal perforation. Specimen pathology confirmed the limbal dermoid tissue in all cases. The surgery resulted in an improved appearing ocular surface in all cases as seen on photographed head shots under natural lighting conditions. The corneal tattoo was clearly appreciated under slit lamp external photographs in bright light only. All patients and family members were satisfied with the cosmetic results. Resolution of patient discomfort and wound epithelialization was achieved by postoperative week 1 or 2 in all cases. The only postoperative complication was the recurrence of a preoperative pseudo-pterygium in one case (Case #3). There were no cases of infection, persistent epithelial defects, wound dehiscence, dellen formation where there previously was none during the follow-up period.

### Secondary outcomes: Visual outcome and refraction

In all patients except two cases, who were too young to obtain a BCVA (Case #15, #21), the mean best corrected visual acuity (BCVA) was 0.22±0.31 logMAR preoperatively and 0.18±0.28 postoperatively without statistical significance (p = 0.231) (Table 2). The mean corneal astigmatism was 2.25±2.45 diopter (D) preoperatively and 1.50±2.58D postoperatively. The mean ocular astigmatism was 2.67±2.09D preoperatively and 2.25±1.39D postoperatively. There was no statistical difference in corneal/ocular astigmatism between preoperatively and postoperatively (p = 0.156 and 0.475, respectively). The mean SE was 0.12±3.19D preoperatively and -0.21±3.02 D postoperatively with a statistically significant difference (p = 0.037).

**Table 2:** Mean change in vision, refraction, and keratometry after dermoid excision, corneal tattooing, and limbal conjunctival autograft.

	Preoperative	Postoperative	Difference	Significance*
BCVA(LogMAR)	0.22±0.31	0.18±0.28	-0.04	0.23
Refractive error (D)	0.67±2.80	0.28±2.63	-0.39	0.10
Ocular astigmatism (D)	2.67±2.09	2.25±1.39	-0.42	0.48
Corneal curvature (D)	42.06±1.63	42.08±1.49	0.02	0.99
Corneal astigmatism (D)	2.25±2.45	1.50±2.58	-0.75	0.16
Mean SE(D)	0.12±3.19	-0.21±3.02	-0.33	0.04

*The Wilcoxon signed-rank test. A p-value< 0.05 was considered statistically significant.

BCVA: Best Corrected Visual Acuity

SE: Spherical Equivalent

### Case

This is a case #8 (B of Fig 2) of an 11-year-old female patient with an inferotemporal dermoid in her right eye (Fig 3A). Her preoperative corrected vision was 20/20 (logMAR 0) in both the eyes. Preoperative refraction was +0.25 Dsph -0.75 Dcyl at 170° in the right eye, and -1.50 Dsph -0.75 Dcyl at 160° in the left eye. After the surgery, her vision remained unchanged for 5 years. When evaluated at 3 years, the refraction in right eye changed to -0.25 Dsph -0.50 Dcyl at 4°, which showed a minimum change without affecting her corrected vision. On postoperative day 1, the grafted conjunctiva was clearly visible (Fig 3B). After 3 weeks, the subconjunctival hemorrhage resolved, and the grafted conjunctival margin was faintly visible (Fig 3C).

No corneal opacity, conjunctival neovascularization, or pseudopterygium was observed at postoperative 3 years (Fig 3D), thus showing good functional and cosmetic results.

## Discussion

In our previous research, we reported a small series of four cases of limbal dermoid treated with excision, corneal tattooing, and sutureless limbal conjunctival autograft, with a short follow-up period [4]. As an extension of the previous research, we report on 24 cases of limbal dermoids treated in the same manner with a follow up period ranging from 41 to 94 months in the present study. There was no significant complication during the period. Our results indicate that this technique is a safe and durable treatment to improve the cosmetic appearance for various grades of limbal dermoid.

Proper cosmesis after limbal dermoid surgery requires the appearance of a white sclera and clear cornea. The conventional way to achieve this with higher grade lesions is to perform a deep excision and replace the defect with a lamellar corneoscleral graft [8–10]. This procedure requires donor tissue and is significantly more complex than simple excision. In addition, it carries the risk of graft failure or rejection. Therefore, this method is not frequently used. As an alternative, corneal tattooing can be used as an adjunct to simple excision to hide the corneal opacity [11, 12]. With only a simple excision of the dermoid, corneal opacity and adjacent conjunctival neovascularization are inevitable, resulting in undesirable cosmesis (Fig 4). We discovered that manufactured black tissue marking dye successfully blended with dark iris of the Asian population in the current study. The dye did not cause any adverse reaction and did not fade over study period [4, 5]. We injected the dye into the anterior stroma. Although there is a single case report of accidental perforation with this technique, we have never experienced this complication [13]. When corneal tattooing is performed very carefully, perforation rarely occurs [4, 5], as the surgeon sees the level of the tip of the needle, and he/she can notice the location of the tip. There are a large number of metallic and organic dyes that can yield a variety of colors. Bandivadekar et al describe the use of gold chloride to create a golden brown to dark brown color that successfully matched the iris color in a group of patients from India [14]. They applied the dye superficially which could be an alternative technique to ours.

As some studies have reported persistent epithelial defects after dermoid excision [8], we have not experienced any persistent corneal epithelial defects, and in all our cases, corneal epithelial healing was completed within 2 weeks. It is possible that the limbal cells relocated from the superior conjunctiva to the lesion. Our technique utilized a limbal conjunctival autograft with fibrin glue. Three of our cases were limbal dermoids with diameters as large as 7mm and we had no instances of persistent epithelial defects or corneal vascularization. There was one case of recurrent pseudopterygium, but there were no cases of new postoperative peripheral corneal vascularization. The autografted limbal cells seemed to prevent the conjunctival growth of the cornea, as limbal cells play a key role as a barrier. Harvesting limbo-conjunctival tissue from the superior limbal conjunctiva is widely used as an auto-limbo-conjunctival graft in many pterygium surgeries [15]. The grafted limbal tissue is believed to prevent or lower the recurrence rate [15]. We got a hint from this point and adjusted this method to our surgery.

**Fig 5:**
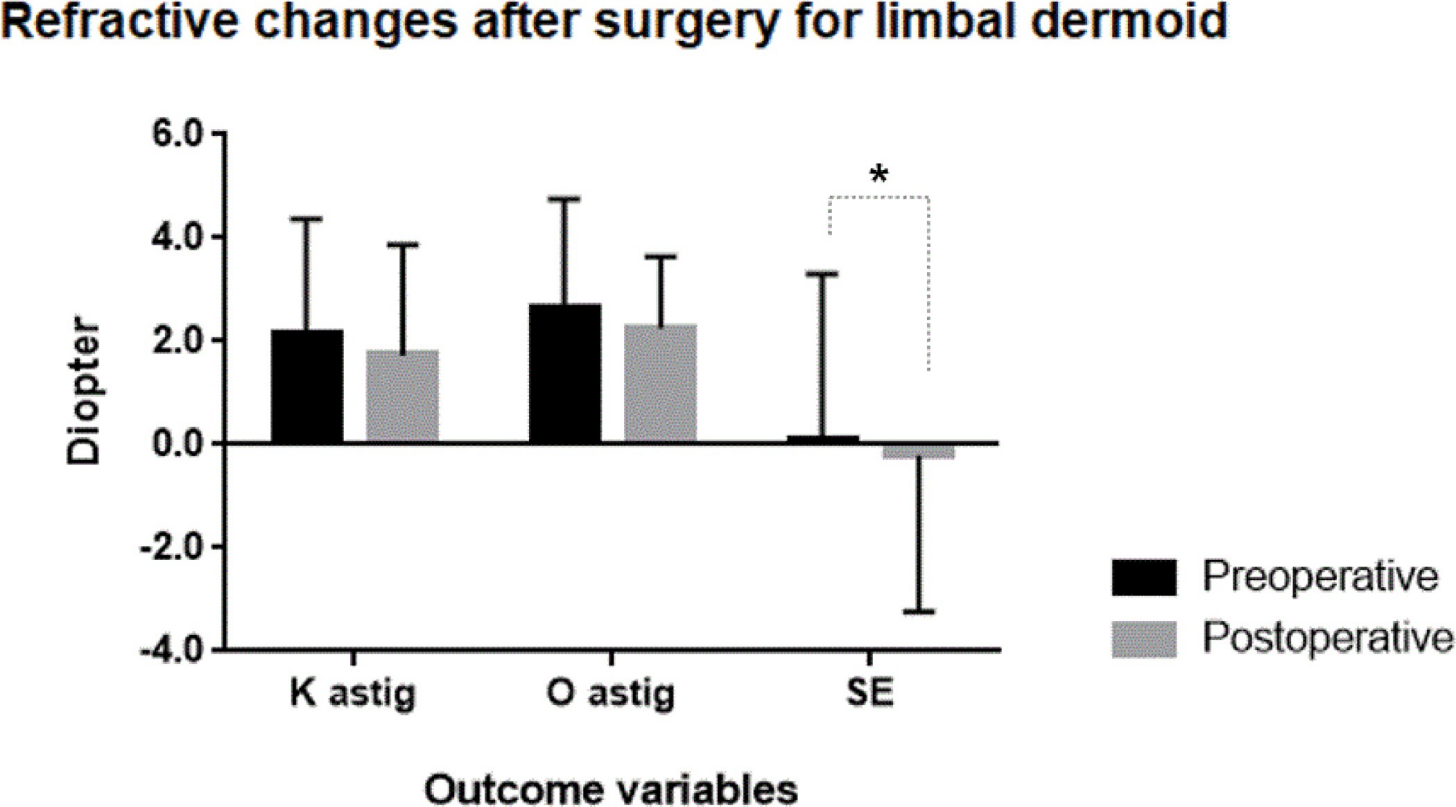
Refractive changes after surgery for limbal dermoid. Asteroid refers to statistical significance (p<0.05). (K astig: corneal astigmatism by auto keratometry, O astig: ocular astigmatism by manifest refraction test, SE: spherical equivalent).

Overall, the initial BCVA was good with a mean of 0.22±0.31 LogMAR. None of the cases developed BCVA worsening after surgery, although there was also no statistically significant improvement in final BCVA. The lack of improvement is probably due to the absent involvement of the visual axis and no statistically significant change in final corneal or refractive astigmatism. Limbal dermoids cause flattening in the meridian of the lesion, however a number of studies have found no significant change in refractive error with only moderate to severe corneal astigmatism [5, 6, 16]. It is hypothesized that the astigmatism may be due to molding of the structure of the corneoscleral wall rather than a compressive force. Interestingly, Shen et al did find improvement in astigmatism within a cohort having extreme preoperative astigmatism (>6D) [6]. If molding of the corneoscleral wall occurs over time, then early surgical intervention may prove beneficial for moderate to severe dermoid induced astigmatism. There were trends for BCVA to be slightly improved from 0.22 logMAR to 0.18 logMAR, and for corneal and ocular astigmatism to be decreased after surgery, which was far less than we expected by the morphological improvement. Interestingly, a slight but statistically significant myopic change of 0.33 diopter was observed (Fig 5). We regard that this may be due to growth process due to 3-year period after surgery. Further studies to verify it are needed.

The current study has a number of limitations. It is retrospective with a relatively small sample size. Some data was missing because patients were too young to perform testing. We did however obtain long-term surgical results in considerable cases when considering the low incidence of limbal dermoids. Further longitudinal studies with a larger sample size are needed to confirm our results. The strength of our study is to extend out previous surgical methods for limbal dermoid to include larger and more severe dermoid cases. In addition, we demonstrated the safety and effectiveness of this novel surgical method during the long-term follow up periods. In particular, it does not require sutures, and prevents future corneal opacity by tattooing, especially helpful for cosmetic outcome. Therefore, we can suggest that it is easily performed in an innovative way and useful for various sizes of limbal dermoid disfiguring cornea, helping mental satisfaction of young patients and parents.

In conclusion, as follow-up research of our previous study introducing the novel surgical method, we reported a favorable result of limbal dermoid which often causes cosmetic concerns for which patients and their families desire definitive treatment for a longer observation period. Based on the results, we can recommend the procedure for patients with non-visual axes involving limbal dermoids, moderate to severe corneal astigmatism, dark irides, and no concomitant pseudo-pterygium. Further studies are needed to determine its efficacy for patients with extreme astigmatism and light colored irides.

## References

[1] Shields CL, Shields JA. Tumors of the conjunctiva and cornea. Surv Ophthalmol. 2004;49: 3–24. doi: 10.1016/j.survophthal.2003.10.008 14711437

[2] Mann I. Developmental Abnormalities of the Eye. 2n ed. Philadelphia, PA: British Medical Association; 1957; 74–91.

[3] Foster JA, Kherani F. Aesthetic Considerations in Pediatric Oculoplastic Surgery. Pediatric Oculoplastic Surgery. 2017; 279–290. doi: 10.1007/978-3-319-60814-3_18

[4] Jeong J, Song Y-J, Jung S-I, Kwon J-W. New Surgical Approach for Limbal Dermoids in Children. Cornea. 2015;34: 720–723. doi: 10.1097/ico.0000000000000440 25881973

[5] Cha DM, Shin K-H, Kim KH, Kwon J-W. Simple keratectomy and corneal tattooing for limbal dermoids: results of a 3-year study. Int J Ophthalmol-chi. 2013;6: 463–6. doi: 10.3980/j.issn.2222-3959.2013.04.10 23991379 PMC3755304

[6] Panton RW, Sugar J. Excision of limbal dermoids. Ophthalmic Surg. 1991;22: 85–9. 2038481

[7] Lang SJ, Böhringer D, Reinhard T. Surgical management of corneal limbal dermoids: retrospective study of different techniques and use of Mitomycin C. Eye. 2014;28: 857–862. doi: 10.1038/eye.2014.112 24858530 PMC4094805

[8] Scott JA, Tan DTH. Therapeutic lamellar keratoplasty for limbal dermoids. Ophthalmology. 2001;108: 1858–1867. doi: 10.1016/s0161-6420(01)00705-9 11581063

[9] Shen Y-D, Chen W-L, Wang I-J, Hou Y-C, Hu F-R. Full-Thickness Central Corneal Grafts in Lamellar Keratoscleroplasty to Treat Limbal Dermoids. Ophthalmology. 2005;112: 1955.e1–1955.e10. doi: 10.1016/j.ophtha.2005.06.015 16168487

[10] Watts P, Michaeli-Cohen A, Abdolell M, Rootman D. Outcome of lamellar keratoplasty for limbal dermoids in children. J Am Assoc Pediatric Ophthalmol Strabismus. 2002;6: 209–215. doi: 10.1067/mpa.2002.124651 12185344

[11] Kaufman A, Medow N, Phillips R, Zaidman G. Treatment of Epibulbar Limbal Dermoids. J Pediat Ophth Strab. 1999;36: 136–140. doi: 10.3928/0191-3913-19990501-11 10358817

[12] Alió JL, Rodriguez AE, Bahrawy ME, Angelov A, Zein G. Keratopigmentation to Change the Apparent Color of the Human Eye. Cornea. 2016;35: 431–437. doi: 10.1097/ico.0000000000000745 26845312

[13] Jalil A, Ivanova T, Bonshek R, Patton N. Unique case of eyeball tattooing leading to ocular penetration and intraocular tattoo pigment deposition. Clin Exp Ophthalmol. 2015;43: 594–596. doi: 10.1111/ceo.12501 25656085

[14] Bandivadekar P, Agarwal T, Temkar S. Shave Excision With Keratopigmentation for Limbal Dermoid. Eye Contact Lens Sci Clin Pract. 2018;44: e7–e9. doi: 10.1097/ICL.0000000000000257 27058832

[15] Hwang HS, Cho KJ, Rand G, Chuck RS, Kwon JW. Optimal size of pterygium excision for limbal conjunctival autograft using fibrin glue in primary pterygia. BMC Ophthalmol. 2018;18: 135. doi: 10.1186/s12886-018-0790-6 29879926 PMC5992752

[16] Robb RM. Astigmatic Refractive Errors Associated With Limbal Dermoids. J Pediat Ophth Strab. 1996;33: 241–243. doi: 10.3928/0191-3913-19960701-08 8827560

